# Anticancer activity of the protein kinase C modulator HMI‐1a3 in 2D and 3D cell culture models of androgen‐responsive and androgen‐unresponsive prostate cancer

**DOI:** 10.1002/2211-5463.12419

**Published:** 2018-04-17

**Authors:** Maria H. Jäntti, Virpi Talman, Kati Räsänen, Ilari Tarvainen, Hannu Koistinen, Raimo K. Tuominen

**Affiliations:** ^1^ Department of Pharmacology and Pharmacotherapy University of Helsinki Finland; ^2^ Department of Clinical Chemistry Medicum University of Helsinki and Helsinki University Hospital Finland

**Keywords:** drug development, prostate cancer, protein kinase C, senescence

## Abstract

Prostate cancer is one of the most common cancers in men. Although it has a relatively high 5‐year survival rate, development of resistance to standard androgen‐deprivation therapy is a significant clinical problem. Therefore, novel therapeutic strategies are urgently needed. The protein kinase C (PKC) family is a putative prostate cancer drug target, but so far no PKC‐targeting drugs are available for clinical use. By contrast to the standard approach of developing PKC inhibitors, we have developed isophthalate derivatives as PKC agonists. In this study, we have characterized the effects of the most potent isophthalate, 5‐(hydroxymethyl)isophthalate 1a3 (HMI‐1a3), on three prostate cancer cell lines (LNCaP, DU145, and PC3) using both 2D and 3D cell culture models. In 2D cell culture, HMI‐1a3 reduced cell viability or proliferation in all cell lines as determined by the metabolic activity of the cells (3‐(4,5‐dimethylthiazol‐2‐yl)‐2,5‐diphenyl‐tetrazolium bromide assay) and thymidine incorporation. However, the mechanism of action in LNCaP cells was different to that in DU145 or PC3 cells. In LNCaP cells, HMI‐1a3 induced a PKC‐dependent activation of caspase 3/7, indicating an apoptotic response, whereas in DU145 and PC3 cells, it induced senescence, which was independent of PKC. This was observed as typical senescent morphology, increased β‐galactosidase activity, and upregulation of the senescence marker p21 and downregulation of E2F transcription factor 1. Using a multicellular spheroid model, we further showed that HMI‐1a3 affects the growth of LNCaP and DU145 cells in a 3D culture, emphasizing its potential as a lead compound for cancer drug development.

**Abbreviations**
DAG: diacylglycerol
DAGK: diacylglycerol kinase
E2F1: E2F transcription factor 1
ERK: extracellular signal regulated kinase
HMI‐1a3: 5‐(hydroxymethyl)isophthalate 1a3
MRCK: myotonic dystrophy kinase‐related Cdc42 binding kinase
PKC: protein kinase c
PMA: phorbol 12‐myristate‐13‐acetate
RasGRP: Ras guanine nucleotide‐releasing protein

Prostate cancer is one of the most common cancer types with estimated 161 000 new diagnoses and more than 26 000 deaths in 2017 in the United States alone 1. Worldwide around 307 000 people died of prostate cancer in 2012, making it the fifth most common cause of cancer death 2. Although the 5‐year survival rate in prostate cancer is high, its prevalence and common development of castration resistance, followed by androgen‐deprivation therapy, is a prominent problem and therefore alternative therapeutic strategies are needed.

One intensively studied target for cancer treatment is protein kinase C (PKC) family of serine/threonine kinases (reviewed in 3). In mammals, 10 PKC isoforms are divided into three groups according to their mode of activation. Classical/conventional PKCs are activated by both Ca^2+^ and diacylglycerol (DAG), novel PKCs require only DAG for activation, and atypical PKCs respond to neither DAG nor Ca^2+^, but require other activators (such as anionic phospholipids). In both conventional and novel PKC subtypes, DAG induces activation by binding to C1‐domain, which is also the binding site of phorbol‐esters, potent PKC activators.

The expression levels and activity of different PKC isoforms have been studied in various types of patient‐derived cancer tissues and cancer cell lines, with partly contradictory results 4, 5. Nevertheless, it has been well established that pharmacological activation of PKC after initiation of tumor formation leads to tumor promotion 6. This observation is based on experiments done using phorbol 12‐myristate‐13‐acetate (PMA), often at high concentrations, as the PKC activator. However, prolonged exposure to high concentrations of PMA leads to dephosphorylation and degradation (i.e., downregulation) of PKC. Furthermore, since all PKC agonists are not tumor promoters, it seems plausible that tumor promotion is not due to PKC activation *per se*, but could instead be related to PKC downregulation and, thus, to the lack of PKC activity. In line with this, a recent study analyzed PKC mutations in a large number of biopsies from various human cancers and found that PKC in fact plays a tumor‐suppressive role 7. In these biopsy samples, none of the observed PKC mutations increased PKC activity, but the cancer‐associated mutations were found to be loss‐of‐function mutations. Interestingly, correcting a PKCβ loss‐of‐function mutation in DLD1 colon cancer cell line using CRISPR/Cas9 technique led to decreased anchorage‐independent growth in soft agar and decreased cell viability in suspension 7. In conclusion, it seems that activation of PKC, without inducing downregulation, could provide a good strategy for treating at least some forms of cancer. The role of PKC (mainly PKCδ) in apoptosis of prostate cancer cells has been shown in several studies 8, 9, 10, 11, 12. In some of these studies, PKC was activated directly with PMA, but also paclitaxel‐induced apoptosis has been shown to require PKCδ 13. Furthermore, a loss of PKCδ expression in high Gleason score prostate tumors has been reported and this was associated with resistance to paclitaxel treatment 14.

Our group has developed small molecule compounds designed to bind to the C1‐domain of PKCs to modulate their activity 15. These isophthalic acid derivatives modulate PKC activity without inducing downregulation of PKC (Fig. S1). We have previously shown that they induce a morphological change (elongation) in HeLa human cervical cancer cells and inhibit their proliferation 16. In order to further explore the potential of isophthalate derivatives in cancer drug development, we elucidated the effects of 5‐(hydroxymethyl)isophthalate 1a3 (HMI‐1a3) in androgen‐responsive and androgen‐unresponsive prostate cancer cell lines, namely LNCaP, DU145, and PC3.

## Materials and methods

### Chemicals

All chemicals used in this study were commercially available, except for the isophthalate derivative HMI‐1a3 (bis(3‐trifluoromethylbenzyl) 5‐(hydroxymethyl)isophthalate) and NI‐15e (bis(3‐trifluoromethylbenzyl) 5‐nitroisophthalate), which were synthesized at the Division of Pharmaceutical Chemistry and Technology, Faculty of Pharmacy, University of Helsinki, as described previously (Boije af Gennäs *et al*. 15). The identity of the compounds was verified with a Bruker Ascend 400 MHz‐Avance III HD NMR spectrometer (Bruker Corporation, Billerica, MA, USA), and the purity of each batch was at least 95% as confirmed by LC‐MS analyses with a Waters Acquity® UPLC system (Waters, Milford, MA, USA) equipped with an Acquity UPLC® BEH C18 column (1.7 μm, 50 × 2.1 mm, Waters, Dublin, Ireland), an Acquity PDA detector and a Waters Synapt G2 HDMS mass spectrometer (Waters, Milford, MA, USA) via an ESI ion source in positive mode. Phorbol 12‐myristate 13‐acetate (PMA), bryostatin‐1, doxorubicin, pan‐caspase inhibitor Q‐VD‐OPH, and the pan‐PKC inhibitor Gö6983 were from Sigma‐Aldrich (Steinheim, Germany). Staurosporine was from Tocris Bioscience (Bristol, UK).

### Cell culture

LNCaP, DU145, and PC3 cells were from ATCC (Manassas, VA, USA) (HTB‐81; CRL‐1740 and CRL‐1435) and were authenticated using microsatellite markers (Promega GenePrint 10 System, Promega, Madison WI, USA) at the Institute for Molecular Medicine Finland FIMM Technology Centre (University of Helsinki, Finland) to confirm their identity. LNCaP and DU145 were cultured in RPMI1640 medium (Cat #1060120, MP Biomedicals, Santa Ana, CA, USA), PC3 cells in Ham's F12K (Kaighn's modification, Cat #21127022; Gibco, Thermo Fisher Scientific, Waltham, MA, USA) medium, and HeLa cells were cultured in Dulbecco's modified Eagle's medium (D‐7777; SIGMA) all of which were supplemented with 10% fetal bovine serum, 100 U·mL^−1^ penicillin, and 100 μg·mL^−1^ streptomycin (all from Gibco). Cell cultures were maintained in a humidified incubator with 5% CO_2_ at 37 °C. All experiments were done in the above‐described cell culture media.

### Cell viability assays

Cell viability was measured using mitochondrial oxidoreductase activity assay [3‐(4,5‐dimethylthiazol‐2‐yl)‐2,5‐diphenyl‐tetrazolium bromide (MTT)], and lactate dehydrogenase (LDH) test was used to measure the amount of LDH released from cells with compromised cell membrane integrity. The cells were plated on 96‐well plates (8000–10 000 cells per well, in serum‐supplemented media) and exposed to the compounds for 24 h, after which LDH assay was carried out using 50 μL samples of cell culture media and MTT assay with the cells. For LDH assay, 50 μL of LDH substrate solution (1.3 mm β‐nicotinamide adenine dinucleotide, 660 μm iodonitrotetrazolium, 54 mm L(+)‐lactic acid, 280 μm phenazine methosulphate (all from Sigma‐Aldrich) in 0.2 m Tris/HCl, pH 8.2) was added to the media samples. After a 30‐min incubation at room temperature (RT), the reaction was stopped by adding 50 μL of 1 m acetic acid and the absorbance was measured at 490 nm. Background absorbance was measured from the wells without cells. Untreated cells were used as controls for spontaneous LDH release, and maximal LDH release was determined from cells lysed with 0.9% Triton X‐100 prior to taking media samples. For the MTT assay, MTT (3‐[4,5‐dimethylthiazol‐2‐yl]‐2,5‐diphenyltetrazolium bromide, from Sigma‐Aldrich) solution was added to the cells at 0.5 mg·mL^−1^. The cells were then incubated in cell culture conditions for 2 h, after which cell culture media was aspirated and replaced with 200 μL DMSO. The absorbance was then measured at 550 nm with absorbance at 650 nm subtracted as background.

### Thymidine incorporation assay

The cells were plated on 6‐well plates (500 000 LNCaP cells per well and 250 000–300 000 PC3 or DU145 cells per well) and 48 (LNCaP) or 24 h after plating exposed to the test compounds for 24 h. For the last 6 h of treatment, [methyl‐^3^H] thymidine (PerkinElmer, Turku, Finland) was added in the incubation medium at 1 μCi·mL^−1^. After incubation, the cells were washed with ice‐cold PBS and free unbound thymidine was precipitated with 5% trichloroacetic acid and discarded. Finally, the cells were lysed with 0.1 m NaOH and the lysates collected to scintillation vials. Optiphase HiSafe scintillation cocktail (PerkinElmer) was added, and the counts were measured with Wallac scintillation counter (PerkinElmer).

### Apoptosis assay

Activation of caspase 3/7 was used to determine whether apoptosis was induced in the cells. Briefly, cells were grown on white‐walled clear bottom 96‐well plates (PerkinElmer) and treated with test compounds as described above for the MTT assay. After a 24‐h treatment, caspase 3/7 activation was determined with Caspase‐Glo 3/7 Assay kit (Promega) according to the manufacturer instructions and the luminescence was quantified using Victor^2^ microplate reader (PerkinElmer).

### Beta‐galactosidase staining and activity measurements

For the β‐galactosidase staining, the cells were seeded on 24‐well plates at 20 000–30 000 cells per well 24 h prior to treatment. The cells were then treated with test compounds for 72 h, fixed, and stained with Senescence β‐galactosidase Staining kit (#9860, Cell Signaling Technology, Danvers, MA, USA) according to the manufacturer's instructions. Briefly, the cells were rinsed once with PBS, fixed with solution provided in the kit for 15 min at RT, and rinsed again two times with PBS. Staining solution was then added to the cells, and the well plate was sealed and incubated for overnight at 37 °C in normal atmosphere. The cells were examined and imaged with Canon EOS 600D camera (Canon, Tokyo, Japan) on Olympus CKX41 inverted microscope (Olympus, Tokyo, Japan) on the following day.

β‐Galactosidase activity was measured utilizing 96‐well Cellular Senescence Assay kit (CBA‐231; Cell Biolabs, San Diego, CA, USA). For this assay, cells were seeded on a 96‐well plate at 2000 cells per well, let to attach overnight, and treated with the compounds for 72 h. After treatment, the cells were rinsed with PBS and lysed. Lysates were centrifuged and the supernatants collected and incubated with fluorescent substrate for 2 h at 37 °C, protected from light. The reaction was then stopped and the fluorescence (Ex 360 nm/Em 465 nm) measured with Victor^2^ microplate reader (PerkinElmer).

### Western blotting

The cells were grown on 6‐well plates and exposed to the compounds for 24 or 48 h after which they were washed with ice‐cold PBS and lysed on ice with lysis buffer (1 mm EDTA, 150 mm NaCl, 0.25% NP‐40, 1% Triton X‐100, 10 mm Tris/HCl, pH 6.8) supplemented with protease and phosphatase inhibitors (Complete and PHOStop, respectively; Roche, Mannheim, Germany). Lysates were centrifuged (13 000 ***g***, 4 min, 4 °C) and the supernatants collected. Equal amounts of protein (10 μg) were subjected to reducing SDS/PAGE and transferred to poly(vinylidene difluoride) membrane. The membranes were blocked either with 5% milk in 0.1% Tween 20 in Tris‐buffered saline (TBST) or 5% BSA in TBST for 1 h at RT, after which they were incubated overnight at 4 °C in a shaker with primary antibodies (all from Cell Signaling Technology, except anti‐GAPDH) against E2F1 (Cat #3742, 1 : 2000), p21 Waf1/Cip1 (Cat#2946, 1 : 1000), cleaved PARP (#9541, 1 : 1000), and GAPDH (sc47724, 1 : 2000, Santa Cruz Biotechnology, Dallas, TX, USA) in blocking buffer. For determining the PKC protein levels in HeLa cells, all primary antibodies were from Santa Cruz Biotechnology PKCα (#8393), PKCβI (#8049), PKCδ (#937) and were used as 1 : 1000 dilution. The experiments were repeated 3 times with 2 parallels. On the following day, the membranes were washed with TBST and incubated with blocking buffer containing HRP‐linked secondary antibody (goat anti‐rabbit, #170‐6515; Bio‐Rad, CA, USA or anti‐mouse IgG #7076S; Cell Signaling Technology) for 1 h at RT. Secondary antibodies were detected with chemiluminescent substrate (SuperSignal West Pico, #34080; Thermo Fisher) utilizing LAS 3000 Imaging System (Fujifilm, Tokyo, Japan). Quantification was carried out by measuring the optical densities of the immunoreactive bands using imagej software (https://imagej.net/Downloads). The optical densities were always first normalized to GAPDH from the same sample and then to the corresponding control (cells treated with the vehicle only) on the same membrane.

### Total Akt and phosphorylated Akt immunoassays

Total Akt and phosphorylated Akt (Ser 473) levels were determined using AlphaLISA Surefire Ultra kits (Cat # ALSU‐PAKT‐B500 and ALSU‐TAKT‐B500; PerkinElmer). Briefly, LNCaP cells were seeded on 96‐well plates (10 000 cells per well) and exposed to compounds 48 h after plating. Some of the cells were first pre‐incubated with the PKC inhibitor Gö6983 for 10 min and then exposed to compounds for 20 min. After treatment cells were washed briefly with PBS and then lysed with 50 μL per well of lysis buffer from the kit on a shaker for 10 min. After that 10 μL of lysates was transferred onto a 384 plate (Alphaplate; PerkinElmer) and 5 μL of acceptor bead mix was added in each well. After a 1‐h incubation at RT, 5 μL of donor bead mix was added, and the plate incubated for further 1 h (RT) and then measured with EnSpire Multimode Plate Reader (Ex 680 nm, Em 615 nm; PerkinElmer).

### 3D cell culture and live/dead assay

LNCaP or DU145 cell was plated at 5000 cells per well on U‐bottom 96‐well plate coated with agarose. The spheroids were allowed to form for 72 h, after which the compounds were added. After a 72‐h compound exposure, live/dead staining (Thermo Fisher) was performed according to the manufacturer's instructions. Briefly, Hoechst 33342, Calcein AM, and Ethidium Dimer‐1 solutions were diluted and mixed in PBS and added to the spheroids in final concentrations of 10, 2, and 4 μm, respectively. The spheroids were then incubated in humidified incubator at 37 °C for 30 min, protected from light. Spheroids were imaged with Hamamatsu Orca‐Flash4.0 V2 sCMOS camera on a Leica DMi8 inverted fluorescent microscope with 20X objective and the following filter sets: Ethidium Dimer‐1 (live cells) ex 494 nm/em 517 nm; Calcein AM (dead cells) ex 528/em 617 nm; and Hoechst (nuclei) ex 350 nm/em 460 nm (Leica, Wetzlar, Germany).

### Statistics

All statistical analyses and determination of relative EC_50_‐values from MTT data were done with graphpad prism version 5.02 for Windows (GraphPad Software, La Jolla, CA, USA, http://www.graphpad.com). Unpaired *t*‐test or one‐way ANOVA followed by Dunnett's test was used for all comparisons as indicated in the figure legends. Control refers to exposure to vehicle (0.1% DMSO), and N refers to the number of independent experiments. Each experiment was repeated at least three times with two or more parallel samples, depending on the experiment, and is indicated in the figure legend.

## Results

### HMI‐1a3 reduces the viability of prostate cancer cell lines in a dose‐dependent manner

**Figure 1:**
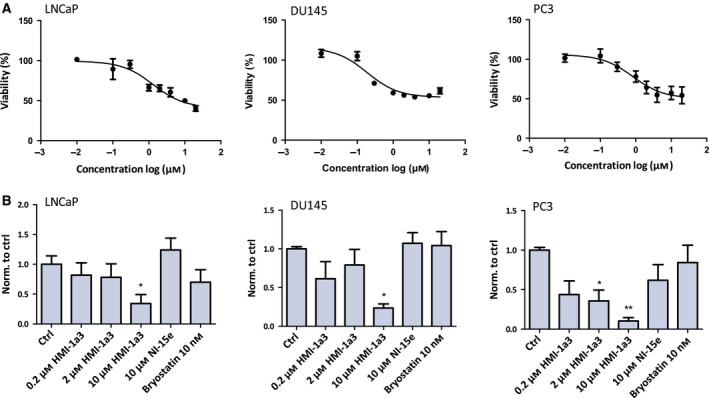
Effects of HMI‐1a3 on viability and proliferation of prostate cancer cell lines. (A) HMI‐1a3 decreases the viability of prostate cancer cells in a concentration‐dependent manner. Cell viability was measured after 24‐h incubation with HMI‐1a3 utilizing MTT assay. The data are presented as mean ± SEM (*N* = 3). (B) The effect of HMI‐1a3, NI15e, and bryostatin on proliferation of prostate cancer cell lines, as measured after 24‐h incubation with compounds using thymidine incorporation assay. The values are presented as mean + SEM (*N* = 3; *P < 0.05; **P < 0.01 vs ctrl, ANOVA followed by Dunnett's test).

The viability of LNCaP, DU145, and PC3 cells decreased dose dependently upon treatment with HMI‐1a3 as determined by the MTT assay (Fig. 1A). The EC_50_ values were as follows: LNCaP 1.06 μm, DU145 0.21 μm, and PC3 1.08 μm. The maximal effect with the highest concentrations was a reduction in viability to around 40% (LNCaP), 60% (DU145), and 55% (PC3). HMI‐1a3 did not induce damage to the cell membranes during the 24‐h incubation in these cells with any of the concentrations as determined by the LDH test (data not shown).

### HMI‐1a3 induces proliferation arrest in all cell lines studied

LNCaP cells show a trend toward an antiproliferative response to HMI‐1a3, when treated for 24 h, as measured with thymidine incorporation assay, but the difference compared to control was not statistically significant with any concentration (Fig. 1B). DU145 cells exhibited an antiproliferative response to HMI‐1a3, but only with 10 μm concentration, whereas PC3 cells exhibited a dose‐dependent antiproliferative response to HMI‐1a3, already 2 μm concentration induced a statistically significant difference in proliferation when compared to control. Compound NI‐15e, which is a structural analog of HMI‐1a3 that does not bind to the C1 domain, had no effect on the proliferation of any of the cell lines. Furthermore, the widely used nontumor‐promoting PKC activator bryostatin‐1 did not affect cell proliferation in any of the cell lines investigated.

### LNCaP cells undergo apoptosis after 24‐h treatment with HMI 1a3

**Figure 2:**
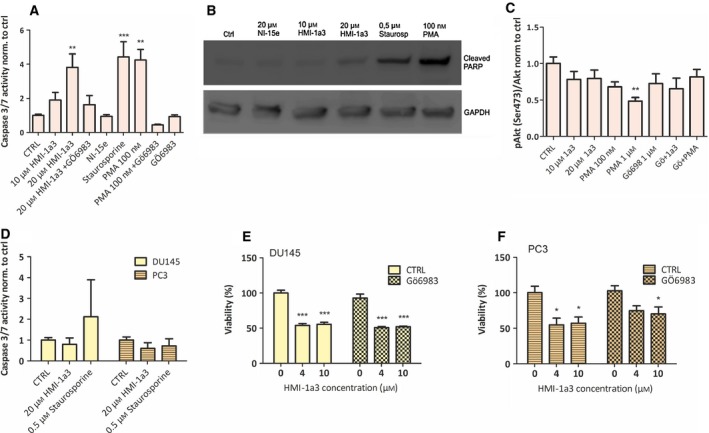
HMI‐1a3 induces PKC‐dependent apoptosis in LNCaP cells and PKC‐independent nonapoptotic reduction in cell viability in DU145 and PC3 cells. (A) Caspase 3/7 activity in LNCaP cells in response to PKC modulators. (B) Apoptosis was verified in LNCaP cells by detecting cleaved PARP with western blotting. Representative blot from three experiments is shown. (C) The proportion of phosphorylated Akt (Ser473) in LNCaP cells in response to PKC modulators. (D) Caspase 3/7 activity in DU145 and PC3 cells in response to HMI‐1a3 and staurosporine. (E,F) The effect of PKC inhibitor Gö6983 (1 μm) on the compromised viability of DU145 (E) and PC3 cells (F) cells. Activity of caspases 3/7 was measured with luminescent substrate and cell viability utilizing MTT assay. Akt phosphorylation was measured with AlphaLISA immunoassay. All quantification data are presented as mean + SEM (*N* = 3; *P < 0.05; **P < 0.01; ***P < 0.001 vs DMSO, ANOVA followed by Dunnett's test).

LNCaP cells have been shown to be directed to apoptosis upon PKC activation 11, 17, 18, 19. We therefore tested whether the HMI‐1a3 induced decrease in cell viability observed with the MTT assay could be due to apoptosis in LNCaP cells. Caspases 3/7 were activated in LNCaP cells following exposure to HMI‐1a3. This seems to be PKC‐dependent, as it was blocked with the PKC inhibitor Gö6983 (Fig. 2A). Furthermore, the level of caspase activation in response to 20 μm HMI‐1a3 was similar to that caused by PMA at 100 nm. However, even 48‐h treatment with HMI‐1a3 does not induce downregulation of PKC isoforms (α, β, and δ) in HeLa cells, whereas 100 nm PMA does (Fig. S1). The inactive isophthalate derivative NI‐15e had no effect on caspase 3/7 activity. The apoptotic response was verified by detecting the appearance of cleaved PARP in LNCaP cells after HMI‐1a3 treatment by western blotting (Fig. 2B).

### Akt phosphorylation status in LNCaP cells is not affected by HMI‐1a3

Protein kinase c activation has been shown to lead to apoptosis of LNCaP cells via dephosphorylation of Akt 19. In LNCaP cells, Akt is constitutively active and phosphorylated because of nonfunctional PTEN 20. We therefore tested whether HMI‐1a3 has any effect on phosphorylation of Ser473 residue of Akt. PMA at 1 μm induced a statistically significant (*P* < 0.01) dephosphorylation of this site, but HMI‐1a3 only slightly decreased the phosphorylation (not statistically significant; Fig. 2C).

### HMI‐1a3‐induced decrease in cell viability in DU145 and PC3 cells does not result from apoptosis and is not mediated by PKC

Because the viability of DU145 and PC3 cells decreases concentration dependently when cells are exposed to HMI‐1a3, we wanted to see whether this was due to apoptosis and whether this response was PKC‐mediated. No apoptosis was detected in DU145 and PC3, as measured by activation of caspases 3/7 (Fig. 2D). In addition, the decrease in cell viability was not affected by the PKC inhibitor Gö6983 (Fig. 2E and 2F) and thus does not seem to be PKC‐dependent.

### PC3 and DU145 cells are driven to senescence upon treatment with HMI‐1a3

**Figure 3:**
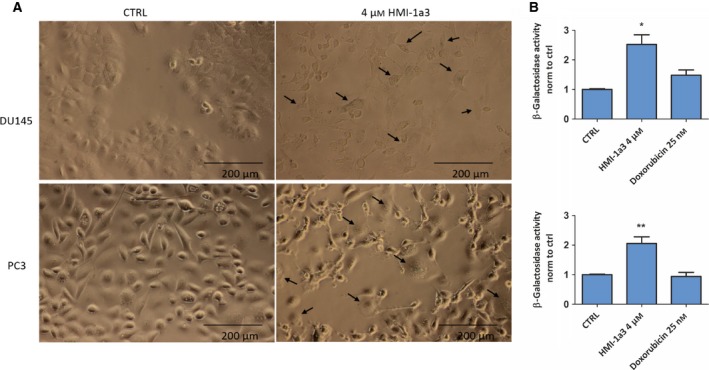
HMI‐1a3 induces senescence in PC3 and DU145 cells. (A) Representative microscopic images (20× magnification) of β‐galactosidase staining in cells exposed to vehicle (DMSO) or 4 μm HMI‐1a3 for 72 h. Senescent cells with distinct morphology and blue staining are indicated with arrows. Scale bars 200 μm. (B) beta‐galactosidase activities quantified using a fluorescent substrate. Data are presented as mean + SEM (*N* = 3; *P < 0.05; **P < 0.01 vs ctrl, ANOVA followed by Dunnett's test).

As the HMI‐1a3‐induced decrease in DU145 and PC3 cell viability did not result from apoptosis, we next went on to characterize the mechanism. In addition to arrest in proliferation, we observed a clear change in the cell morphology upon exposure to HMI‐1a3 in both cell lines, the cells resembling senescent cells with flattened appearance and granulated nuclei. We therefore stained the cells with a senescence marker β‐galactosidase, which confirmed that a proportion of the cells were indeed senescent (Fig. 3A). Furthermore, 4 μm of HMI‐1a3 induced over twofold increase in β‐galactosidase activity (*P* < 0.05) in both cell lines (Fig. 3A,B). Doxorubicin, which has been shown to induce senescence in DU145 and PC3 cells when used in low concentration 21, did not increase β‐galactosidase activity under these conditions.

**Figure 4:**
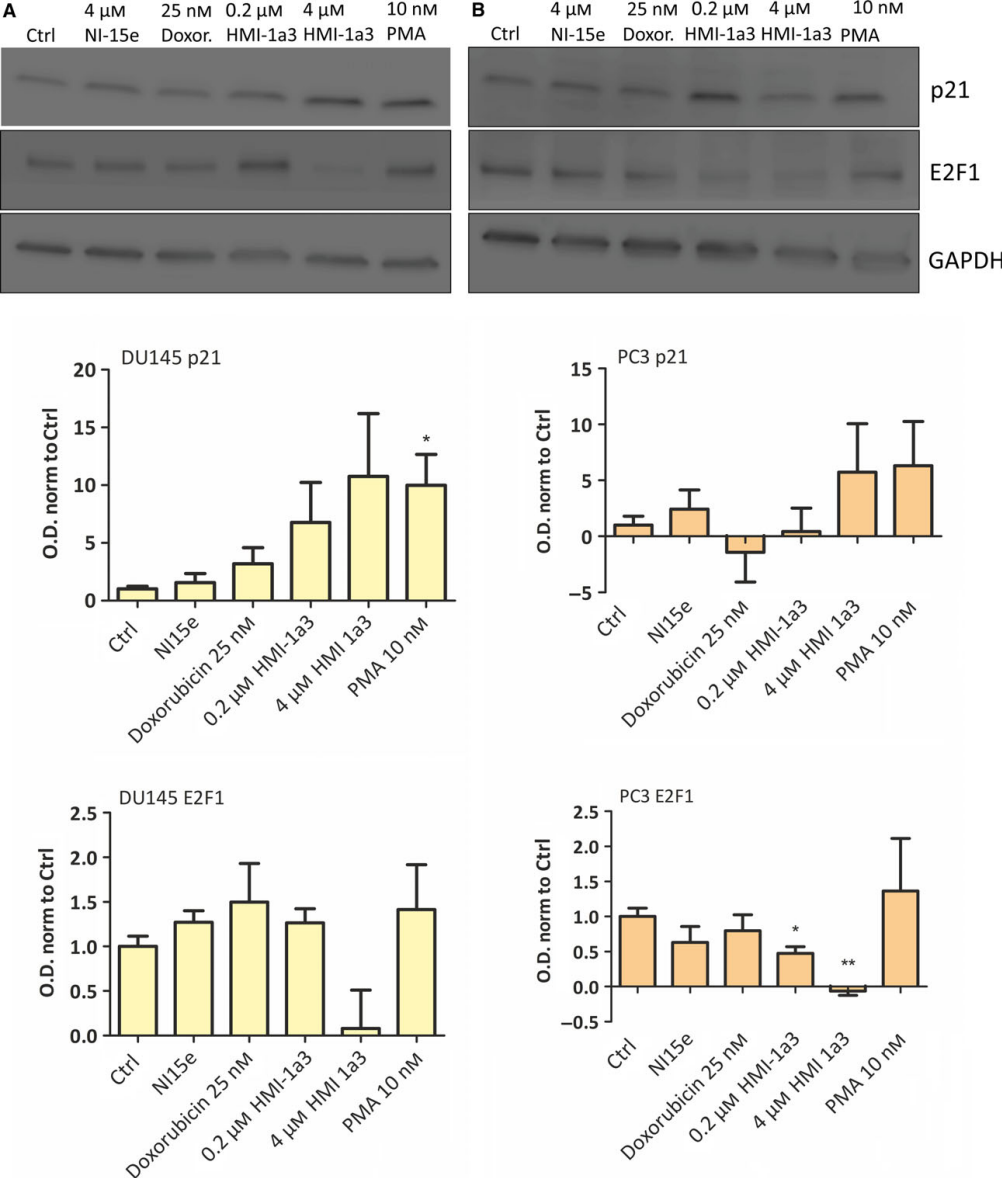
Levels of p21 and E2F1 in DU145 and in PC3 prostate cancer cell lines after 24‐h (DU145) or 48‐h (PC3) treatment. (A,B) Representative western blot images and quantifications from DU145 cells (A) and PC3 cells (B). Data are presented as mean + SEM (*N* = 3; *P < 0.05; **P < 0.01 vs ctrl, ANOVA followed by Dunnet's test).

Finally, to confirm the senescence response, we studied the levels of p21 and E2F transcription factor 1 (E2F1). The level of p21 is increased and the level of E2F1 decreased when cells enter senescence. There was a trend for increased levels of p21 in PC3 cells after a 48‐h treatment and in DU145 cells after a 24‐h treatment (Fig. 4), but the changes were not statistically significant. Correspondingly, the levels of E2F1 tended to decrease in DU145 cells (not statistically significant) and decreased (*P* < 0.01) in PC3 cells (Fig. 4), further confirming that the cells indeed became senescent. NI‐15e or doxorubicin had no effect on the levels of p21 or E2F1. PMA had a similar effect on p21 as HMI‐1a3, but no effect on E2F1 levels.

### HMI‐1a3 increases the number of dead cells in DU145 spheroids and affects the geometry of LNCaP cells in 3D cell culture

**Figure 5:**
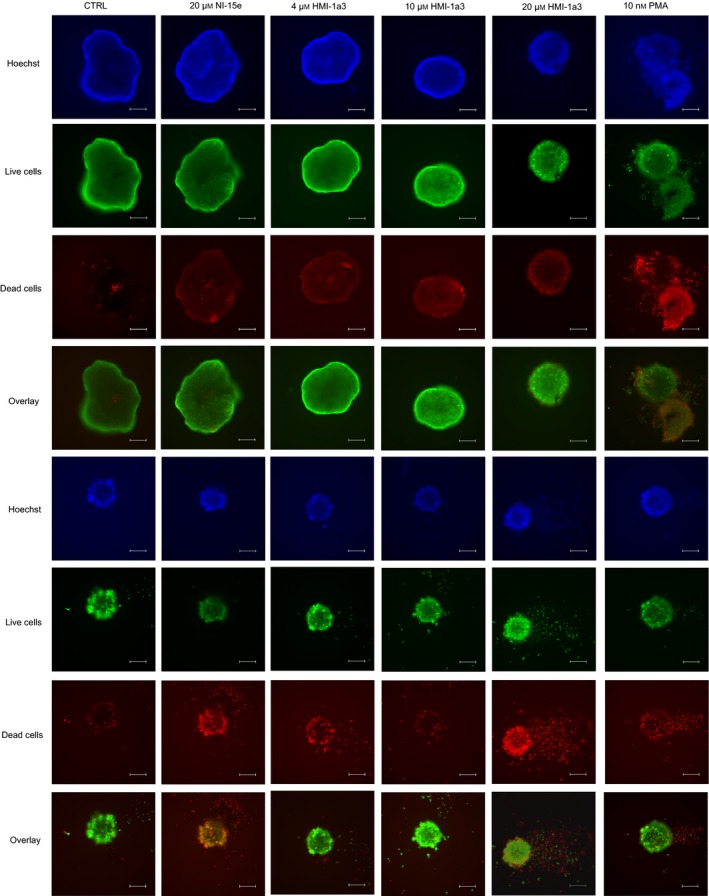
Effects of PKC agonists on prostate cancer cell growth in 3D spheroid model. Representative microscopic images of live‐dead‐stained spheroids of (A) LNCaP and (B) DU145 cells after 72‐h exposure to 20 μm NI‐15e, 4, 10, and 20 μm HMI‐1a3, and 10 nm PMA as indicated. The cells were imaged with Leica DMi8 inverted fluorescent microscope using 10× magnification. The experiments were repeated at least three times with three replicates for each condition. Scale bars, 200 μm.

As the cell culture techniques evolve, it has become more and more pronounced that especially in cancer research the experimental models should mimic the physiological situation as much as possible. For cancer cells, the cell–cell contacts are of utmost importance. While in two‐dimensional (2D) models, the cell–cell contacts do not accurately reflect the situation *in vivo*, 3D cell culture systems recapitulate the physiological situation more closely 22. Therefore, we wanted to see whether HMI‐1a3 has an effect on prostate cancer cells also in a 3D context. The LNCaP spheroids appeared to decrease in size and became more round and compact in a concentration‐dependent manner (Fig. 5A). The negative control compound NI‐15e had no effect on the spheroid shape, whereas PMA induced similar rounding of spheroids as HMI‐1a3. The amount of live and dead cells was visualized using Live/Dead staining, and while NI‐15e had no effect on the quantity of dead cells, PMA seemed to induce cell death. In DU145 spheroids, the number of dead cells increased with increasing HMI‐1a3 concentration (Fig. 5B). PC3 cells do not form compact spheroids in this model and therefore were not studied.

## Discussion

In search of new drug targets for prostate cancer, PKC has been in focus for decades. The levels of different PKC isoforms in prostate cancer have been studied 4 and both inhibition and activation of PKC have been suggested as promising strategies for treating prostate cancer 8, 14, 19, 23, 24. The recent comprehensive analysis of cancer‐associated mutations in PKC isoforms indicates that the role of PKCs is in fact tumor‐suppressive 7. This explains at least partially the failures in PKC‐targeted cancer drug discovery, as the efforts by pharmaceutical companies have mainly been directed toward developing PKC inhibitors. Of note, the only PKC‐targeted drug accepted for clinical use is ingenol mebutate, a PKC activator used for the treatment of actinic keratosis (preliminary stage of skin cancer) 25. Development of activators, which do not induce downregulation of PKC protein levels (and thereby reduction in activity), thus seems to be the most promising strategy for PKC‐targeted cancer drug discovery. PKC maturation involves phosphorylation of several sites, for example, activation loop. These mature PKCs reside in the cytoplasm and are recruited to the plasma membrane only in the presence of activators such as DAG or phorbol‐esters (reviewed in 3). However, in the membrane‐bound active state PKC is sensitive to dephosphorylation followed by proteolysis by endosomes or ubiquitin pathway and therefore prolonged activation leads to decrease in PKC protein levels (reviewed in 3).

We have previously shown that the isophthalate derivative HMI‐1a3, which binds to the C1 domain of PKCs and activates PKCs in cellular context without inducing downregulation, inhibits the proliferation of HeLa cells 16. In the present study, we aimed to characterize the effects of the isophthalate‐structured PKC modulator HMI‐1a3 on prostate cancer cells *in vitro*. The prostate cancer cell lines used in this study represent different types of prostate cancer: DU145 and PC3 cells are androgen‐unresponsive cells, whereas LNCaP cells are androgen‐responsive. These cell lines also differ from each other in relation to PTEN and p53 expressions. DU145 express functional PTEN, but mutated p53. PC3 cells are both PTEN and p53 null, and LNCaP cells do not express functional PTEN, but express wild‐type p53 20, 26, 27.

Although the role of PKC in prostate cancer has been somewhat debated 4, 5, what seems to be clear based on various studies is that PMA‐induced PKC activation induces apoptosis in LNCaP cells 12, 17, 18, 19. In agreement with this, we report here a strong, PKC‐dependent apoptotic response in LNCaP cells upon treatment with HMI‐1a3, which was attenuated with the pan‐PKC inhibitor Gö6983. Furthermore, the level of caspase 3/7 activation in response to HMI‐1a3 was comparable to that induced by the potent PKC activator PMA or staurosporine, which is a widely used inducer of apoptosis. Several mechanisms for phorbol ester‐induced apoptosis in LNCaP cells have been suggested, including dephosphorylation of Akt, activation of p38‐ and PKC‐mediated secretion of death factors (TNFα and TRAIL) 12, 19. However, the mechanism of HMI‐1a3‐induced apoptosis does not seem to involve Akt dephosphorylation. It cannot, however, be completely ruled out, since there is a slight trend toward Akt dephosphorylation when LNCaP cells were treated with HMI‐1a3. Whether the secretion of death factors could explain the effect remains to be studied.

The most problematic cases of prostate cancer are androgen‐independent and resistant to the most common prostate cancer treatment, androgen‐deprivation therapy. As *in vitro* models of this clinically challenging prostate cancer type we used the androgen‐unresponsive cell lines PC3 and DU145. PKC activation does not induce apoptosis in these cells, but instead treatment with HMI‐1a3 drove these cells to senescence. This was verified by β‐galactosidase staining and quantification of β‐galactosidase activity. Furthermore, the level of p21 protein was increased and E2F1 protein level was decreased. Taken together, these changes confirm that the cells were indeed senescent. Although senescence has its unfavorable effects, it has been shown that some currently used anticancer therapies, such as radiation therapy or PKC activator ingenol‐3‐angelate (ingenol mebutate), drive the cancer cells to senescence and this therapy‐induced senescence is considered as one strategy for cancer therapy 28, 29, 30. However, it seems that the HMI‐1a3‐induced senescence was not mediated by PKC, as the PKC inhibitor Gö6983 did not block the effect of HMI‐1a3 in the MTT assay. This result is in line with our previously published data in HeLa cells 16, 31. In these cells, the effect of HMI‐1a3 was not altered by pharmacological PKC inhibition or knockdown of PKCα or PKCδ 31, but required binding to the PKC C1 domain. Other groups have previously shown that in melanoma, breast and colon cancer cells, PKC activation induces senescence via extracellular signal regulated kinase (ERK) hyperactivation 32, 33. These studies were done using C1 domain binding diterpene esters as PKC activators. However, some of the anticancer effects of these compounds have also been attributed to their ability to activate Ras guanine nucleotide‐releasing protein (RasGRP) 34. Therefore, it is plausible that the effects of HMI‐1a3 in PC3 and DU145 cells might be due to other C1 domain containing proteins such as RasGRP. In our earlier studies, we were able to show that the isophthalates bind to other DAG‐responsive C1 domains, which are present in six other protein families: chimaerins, Munc13, myotonic dystrophy kinase‐related Cdc42 binding kinase (MRCK), protein kinase D, RasGRP, and DAG kinase (DAGK) 31, 35, 36. Unfortunately, very little is known about these other C1 domain containing proteins in senescence.

As the isophthalate HMI‐1a3 induced senescence or apoptosis in the three prostate cancer cell lines used in a traditional 2D cell culture system, we wanted to investigate its effects on DU145 and LNCaP cells in a 3D cell culture model. This 3D model is considered (patho)physiologically more relevant, since the cell–cell contacts resemble those within a tissue better than the plastic 2D surfaces 22. Also, it has been shown that cells respond to drugs in different ways in 2D and 3D culture systems and are more sensitive to treatment in 2D models. Furthermore, the low success rates in development of new pharmacological anticancer therapies have been attributed to the use of less physiological 2D models in drug screening 37. In this respect, the effect observed with HMI‐1a3 in 3D cultures of DU145 and LNCaP cells is indeed encouraging. Although the effect was not as dramatic as with PMA, it highlights the potential of C1 domain‐targeting compounds as a therapeutic strategy for cancer.

Taken together, our results indicate that PKC agonists may have potential as prostate cancer drugs. This is important for the future development of adjuvant therapies of castration‐resistant and chemotherapy‐resistant cancers.

## Author contributions

MHJ, VT, KR, HK, and RKT designed the experiments, MHJ, KR, and IT conducted the experiments; MHJ analyzed the data and wrote the manuscript; RKT led the project. All authors critically revised the manuscript and approved the final version.

## Supporting information


**Fig. S1.** Levels of different PKC isoforms in HeLa cells following 24 or 48‐h exposure to PMA or HMI‐1a3. Data is presented as mean +SEM (N=3; *P < 0.05; **P < 0.01 vs ctrl, ANOVA followed by Dunnet’s test).
