# Supplementary figures and images for: Anticancer activity of the protein kinase C modulator HMI‐1a3 in 2D and 3D cell culture models of androgen‐responsive and androgen‐unresponsive prostate cancer
Source: FEBS Open Bio. 2018 Apr 17;8(5):817–28. doi: 10.1002/2211-5463.12419 (PMC5929934; doi:10.1002/2211-5463.12419)

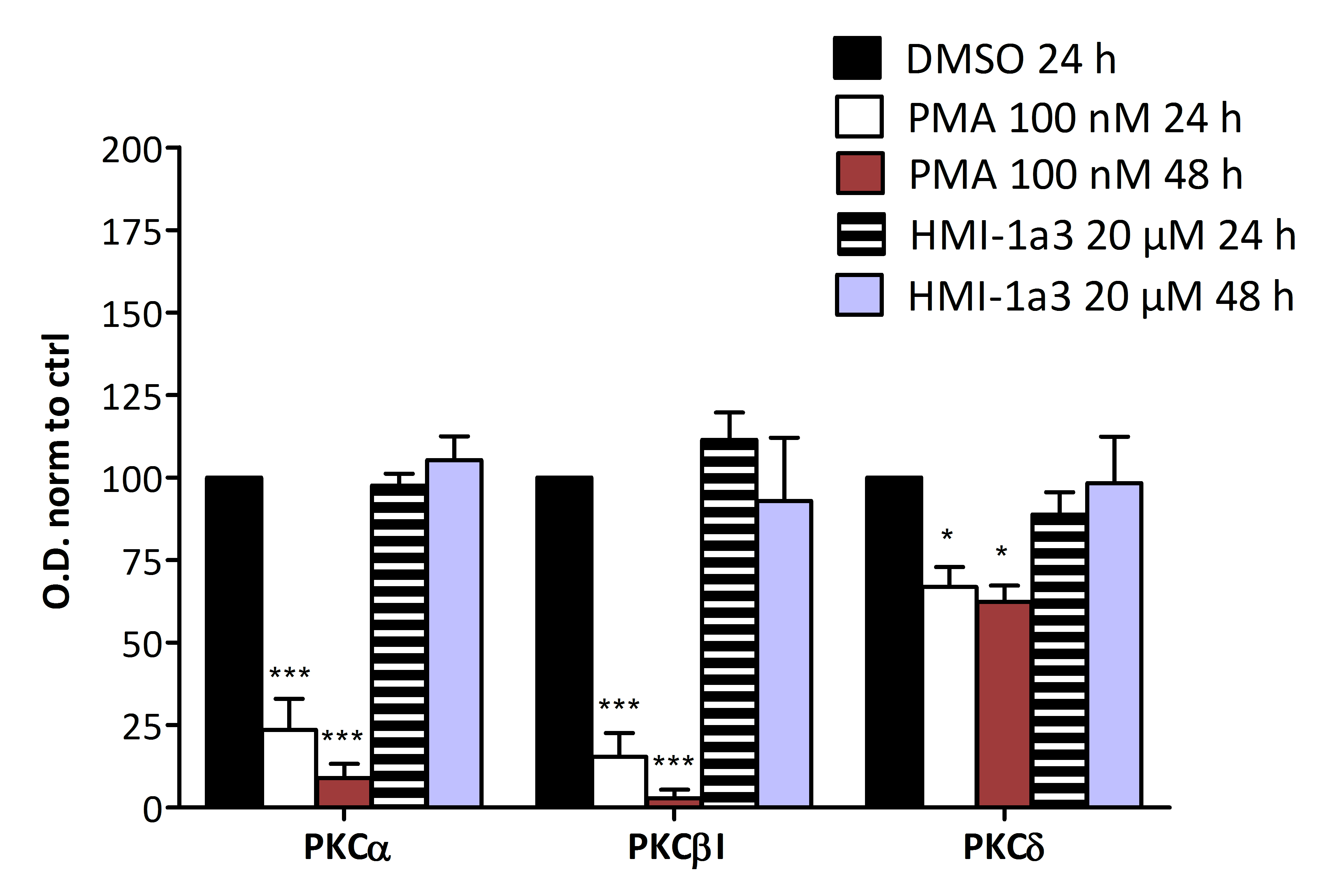

Supplement: Supplementary file 1 — Fig. S1. Levels of different PKC isoforms in HeLa cells following 24 or 48‐h exposure to PMA or HMI‐1a3. Data is presented as mean +SEM (N=3; *P < 0.05; **P < 0.01 vs ctrl, ANOVA followed by Dunnet’s test). [file FEB4-8-817-s001.tif]
